# Evaluation des Connaissances-Attitudes-Pratiques des populations des districts sanitaires de Benoye, Laoukassy, Moundou et N’Djaména Sud sur la rage canine au Tchad

**DOI:** 10.11604/pamj.2017.27.24.11464

**Published:** 2017-05-10

**Authors:** Rolande Mindekem, Monique Lechenne, Idriss Oumar Alfaroukh, Daugla Doumagoum Moto, Jakob Zinsstag, Laurent Tinoaga Ouedraogo, Sahidou Salifou

**Affiliations:** 1 Ministère de la Santé Publique, Tchad; 2 Centre de Support en Santé Internationale, Tchad; 3 Swiss Tropical and Public Health Institute, Bâle, Suisse; 4 Université de Bâle, Suisse; 5 Coordination Régionale de la Composante Santé Animale du Projet d’Appui au Pastoralisme au Sahel, Bamako, Mali; 6 Institut Régional de Santé Publique, Ouidah, Bénin; 7 Université d’Abomey Calavi, Bénin

**Keywords:** Connaissances-Attitudes-Pratiques, ménages, rage, districts sanitaires, Tchad, Knowledge-Attitudes-Practices, households, rabies, health districts, Chad

## Abstract

**Introduction:**

La rage canine demeure une préoccupation en Afrique comme au Tchad. La présente étude vise à évaluer les Connaissances-Attitudes-Pratiques des populations pour la prise en charge appropriée des personnes exposées et une lutte efficace.

**Méthodes:**

C’était une étude transversale descriptive réalisée en juillet et septembre dans quatre districts sanitaires au Tchad en 2015. Les données ont été collectées à l’aide d’un questionnaire auprès des ménages recrutés suivant un sondage aléatoire à 3 degrés.

**Résultats:**

C’était 2428 personnes enquêtées avec un niveau maximum primaire (54,12%). L’âge moyen était de 36 ± 13,50 ans. Ils étaient cultivateurs (35,17%), commerçants (18,04%), ménagères (12,81%). La rage était définie comme une maladie transmise du chien à l’homme (41,43%), une altération du cerveau (41,27%), une sous-alimentation (10,26%). Le chat était faiblement connu réservoir (13,84%) et vecteur (19,77%) ainsi que la griffure comme moyen de transmission (4,61%) et la vaccination du chat comme mesure préventive (0,49%). Les premiers soins en cas de morsure à domicile étaient les pratiques traditionnelles (47,69%), le lavage des plaies (19,48%) ou aucune action entreprise (20,43%). Les ménages consultaient la santé humaine (78,50%), la santé animale (5,35%) et les guérisseurs traditionnels (27%).

**Conclusion:**

La communication en rapport avec des premiers soins à la maison en cas de morsure, la connaissance du chat comme réservoir et vecteur, celle de la griffure comme moyen de transmission et la promotion de la consultation des services vétérinaires en cas de morsure sont nécessaires.

## Abstract (en)

**Introduction:**

Canine rabies remains a concern in Africa as well as in Chad. Our study aimed to evaluate the knowledge, attitudes and practices of the populations towards the appropriate management of people exposed to canine rabies and effective fight against it.

**Methods:**

We conducted a cross-sectional, descriptive study in four health districts in Chad in July and in September 2015. Data were collected from households recruited by three-stage random sampling by means of a questionnaire.

**Results:**

We conducted a survey of 2428 individuals having completed at least primary education level (54,12%). The average age was 36 ± 13.50 years. Surveyed individuals were farmers (35,17%), merchants (18,04%), households (12.81%). Rabies was defined as a disease transmitted from the dog to the man (41.43%), an alteration in brain function (41.27%), an undernourishment (10.26%). The cat was little-known to be a reservoir(13.84%) and a vector (19,77%) as well as licking was little-known to be a transmission medium (4.61%) and cat vaccination to be a preventive measure (0.49%). First aid for a bite at home was the traditional practice (47,69%), wounds washing (19.48%) or no action undertaken (20.43%). Households consulted the Health Service (78.50%), the Animal Health Service (5.35%) and the traditional healers (27%).

**Conclusion:**

A communication campaing for implementing first aid at home in the event of a bite, knowledge about the cat as a reservoir and a vector and licking as a transmission medium as well as the promotion of the consultation of veterinary services in the event of a bite are necessary.

## Introduction

La rage est une maladie infectieuse virale connue depuis l’antiquité mais figure à ce jour parmi les maladies tropicales négligées [1]. Le virus de la rage infecte tous les mammifères à sang chaud. Les cas de rage humaine sont à 90% consécutifs à une morsure de chien [2]. La rage tue environ 60 000 à 70000 personnes dans le monde et 99% de ces cas sont dans les pays à faible revenu notamment en Asie et en Afrique où 40% des victimes sont les enfants de moins de 15 ans [3, 4]. Elle est une menace potentielle pour 3,3 milliards de personnes dans 100 pays [5]. La maladie se présente sous la forme furieuse et paralytique. La prévention de la maladie nécessite la connaissance appropriée du réservoir, du vecteur, du mode de contamination, et aussi des attitudes et pratiques appropriés à adopter en cas de morsure. La plupart des études sont réalisées dans les centres urbains excluant les populations rurales qui sont les plus exposées à cause de l’inaccessibilité géographique des structures, de la PPE et de la faible information sur la rage. L’évaluation des Connaissances-Attitudes-Pratiques (CAP) des populations de ces zones constitue un aspect important pour le contrôle de la maladie. Au Tchad, la rage est endémique et la première évaluation des Connaissances-Attitudes-Pratiques des populations au sujet de la rage dans la ville de N’Djamena a été faite il y a plus de 10 ans dans la ville de N’Djamena [6]. Peu des données sur les Connaissances-Attitudes-Pratiques des populations sont disponibles. L’objectif de l’étude était d’évaluer les connaissances, les attitudes et pratiques des populations relatives à la rage pour contribuer à l’élaboration des stratégies d’intervention adaptées.

## Méthodes

### Sites et population d’étude

Quatre districts étaient sélectionnés dans les Délégations Sanitaires Régionales du Logone Occidental et celle de N’Djamena. La ville de N’Djamena a une superficie d’environ 52 000 hectares et constituée de 59 quartiers répartis dans 10 communes. En 2014, sa population était estimée à 1 203 594 habitants sur la base des données du Recensement Général de la Population et de l’Habitat de 2009 [7]. Le District Sanitaire de N’Djamena Sud est constitué 19 quartiers répartis dans trois communes et a une population estimée à 391 613 habitants en 2014. La Délégation Sanitaire Régionale du Logone Occidental est composée Les District Sanitaire du Laoukassy, Benoye et Moundou qui couvrent une superficie de 8 916 km^2^ et une population de 2014 estimée à 783 400 habitants sur la base des données du Recensement Général de la Population et de l’Habitat [7].

**La population cible de l’étude**: la population cible était le chef de ménage (homme ou femme) ou une autre personne mandatée ayant plus de 18 ans et capable de répondre aux questions.

### Type d’étude, méthode et technique d’échantillonnage

C’était une étude transversale descriptive auprès des ménages recrutés suivant un sondage à trois degrés. Au premier degré, La Délégation Sanitaire Régionale de N’Djamena était incluse dans l’étude par sa position de ville capitale et celle du Logone Occidental était choisie après un tirage au sort sans remise des régions qui ne sont pas contiguës à celle de N’Djamena. Au deuxième degré, le District Sanitaire de N’Djamena Sud a été retenu suivant un choix aléatoire simple et les trois districts du Logone Occidental de manière exhaustive dans l’étude. Les districts considérés comme urbain sont ceux des chef des Délégations Sanitaires Régionales et ce sont Moundou et N’Djamena Sud. Ceux de Bénoye et Laoukassy sont considérés comme des Districts Sanitaires ruraux. Après le choix des Districts Sanitaires, les carrés ont été choisis après avoir dressé l’effectif de la population de chaque District par carré et calculé la population de 2014 le cumul de cette population. Dans chaque Districts Sanitaires, 40 carrés ont été choisis en divisant la population cumulée par le nombre des carrés retenus pour avoir le pas de sondage (k). Ensuite nous avons choisi un chiffre de façon aléatoire entre 1 et k et l’avons appelé « origine choisie au hasard ». Le premier carré inclus dans l’étude est celui dont le chiffre de la population était inférieur ou égal au chiffre de l’origine choisie. Le second carré était celui dont le chiffre de la population est inférieur ou égal à la somme du premier carré inclus dans l’étude et du pas de sondage. Du 3^ème^au 40^ème^ carré, concernaient les carrés dont la population est issue de la somme du chiffre du carré précédent et du chiffre du pas de sondage. Le chiffre du 40^ème^ carré doit être inférieur ou égal au chiffre de la population cumulée du district concerné. C’était dans ces carrés que les ménages ont été choisis de façon aléatoire et enquêtés. La taille de l’échantillon a été calculée sur la base du nombre de chien par ménage (nombre obtenu sur la base du ratio chien/Homme de N’Djamena de l’étude de 2001 de l’intervalle de confiance, de la précision désirée. Nous avons aussi tenons compte du taux de réponses dans le calcul de l’échantillon. La taille de notre échantillon par District sanitaire était de 607 ménages, soit un total de 2428 ménages pour les quatre districts sanitaires retenus.

### La technique de collecte et le questionnaire

La technique de collecte des données auprès des ménages a été l’entretien dirigé et l’outil était un questionnaire structuré suivant les lignes directives de l’OMS [8]. Pour cette étude, les données collectées ont porté sur les caractéristiques socio-démographiques des personnes interviewées, leurs connaissances sur le réservoir, le vecteur du virus rabique ainsi que le mode de transmission de la rage. Elles étaient aussi relatives à la connaissance des signes de la rage humaine et animale, du coût de la prévention de la rage et du traitement antirabique ainsi que les variables liées au comportement adopté en cas de morsure. L’étude pour les ménages qui élèvent au moins un chien qui fait l’objet d’un article à part.

### Recrutement, formation des enquêteurs et pré-test

La formation a concerné 15 enquêteurs parmi lesquels 12 ont participé à la collecte des données et 3 étaient de réserve. Ils avaient au moins le niveau de la licence et possédaient une expérience dans la collecte des données de type Connaissances-Attitudes-Pratiques. Leur formation a été réalisée avec l’appui de formateurs de l’Université de Moundou. A l’issue de la formation, le questionnaire a été testé sur des sites qui n’étaient pas concernés par l’enquête principale. Le pré-test a permis d’aménager certaines modalités avant de la finaliser et l’utiliser.

### Collecte, saisie, traitement et analyse des données

La collecte des données a été réalisée suivant le plan de collecte journalier établi pendant les mois de juillet et septembre et était faite après toutes les formalités auprès des responsables administratifs et locaux. Dans certains cas, les questionnaires ont été administrés en langue locale. Au cours de la collecte, les questionnaires ont été vérifiés manuellement. A la fin de la collecte, les données ont été saisies en double sous Epi Info. 7. La double saisie a été validée. Les statistiques descriptives ont été réalisées en utilisant Epi info. 7. Les résultats étaient produits en pourcentage ou en moyenne avec leur écart type.

### Considérations éthiques

L’étude a reçu l’approbation des Ministères de la Santé Publique, de l’enseignement supérieur et de la recherche scientifique, du développement Pastoral et de la Production Animale et des Mairies. Chaque participant à l’étude a reçu l’information éclairée relative à l’étude et était libre de donner son consentement pour participer à l’étude. L’administration des questionnaires a été faite de façon anonyme et confidentielle.

## Résultats

### Caractéristiques sociodémographiques

Les personnes enquêtées au nombre de 2428 personnes dont plus de la moitié était de sexe masculin et des chefs de ménage. L’âge moyen des enquêtés de 36 ± 13,50 ans. La plupart était de niveau maximum primaire (54,12%) et des chrétiens (87%). La majorité habitait dans des concessions non clôturées et clôturés sans portail fermé. Au sein de ces ménages, 19 003 personnes ont été dénombrées pour une taille moyenne de 7,8 ± 5,1 personnes/ménages. 45,55% des ménages étaient identifiés ayant au moins un chien et il y avait 1,66 chiens/ménage. Les détails des caractéristiques socio-démographiques des personnes enquêtées sont présentés dans le Tableau 1.

**Tableau 1:** Caractéristiques sociodémographiques des personnes enquêtées, résultats de l’étude réalisée dans quatre districts sanitaires au Tchad, 2015

Variables	Bénoye^a^	Laoukassy^a^	Moundou^a^	N’Djaména^a^	Global^b^
**Sexe (%)**					
Masculin	75,29	73,64	51,24	62,77	65,61
Féminin	24,71	26,36	48,76	37,73	34,39
Age moyen des enquêtés (*années)*	38,30	35,46	35,00	35,25	36,00
**Religion (%)**					
Chrétienne	97,36	92,75	95,06	72,31	89,37
Musulmane	0,83	5,60	4,78	27,03	9,56
animiste	1,81	1,65	0,16	0,66	1,07
**Niveau d'instruction (%)**					
Non alphabétisé	30,55	31,96	14,66	10,71	22,08
Primaire	39,21	38,72	31,47	18,78	32,04
Secondaire	27,21	24,38	41,68	33,28	31,80
Supérieur	2,14	3,95	9,23	29,49	11,12
Ecole coranique	0	0,99	2,96	6,92	2,72
Ecole Franco-Arabe	0,89	0	0	0,82	0,25
**Position dans le ménage**					
Chef de ménage	77,03	72,32	47,45	55,45	63,10
Femme du chef de ménage	16,21	20,1	34,10	24,88	24,84
Enfant du chef de ménage	6,10	6,92	14,00	15,48	11,63
autre (parent du chef de ménage ou de sa femme)	0,66	0,66	4,45	4,45	2,43
**Situation des concessions**					
Concessions fermées	5,93	2,47	41,02	53,21	25,66
concessions non fermées	94,07	97,53	58,98	46,79	74,34

a Effectif n=607

b Effectif total N=2428

### Connaissances de la rage, de l’animal réservoir et vecteur du virus rabique et du mode de transmission du virus rabique

La rage était décrite comme une maladie du chien transmise à l’Homme (41,43%), une folie/maladie du cerveau/crise mentale à proportion égale (41%), la malnutrition (10%) et aussi comme l’empoisonnement (1,61%). 6% des enquêtés étaient incapables de fournir une description. Les différents noms locaux pour identifier la rage sont : *« djala », « kor », « wey », « konné », « boula », «djaham »* et tous signifient une altération du cerveau, une folie. La majorité des enquêtés percevaient que le chien était le principal réservoir (96,54%) et vecteur (97,24%) du virus rabique dans une moindre mesure le chat était cité réservoir (13,81%) et vecteur (18,33%). Cependant 3% d’entre eux ne connaissaient aucun réservoir ni vecteur. Le moyen de transmission de la rage le plu connu était la morsure (99%) suivie de la griffure (6%). Peu d’enquêtés étaient capables de citer à la fois deux réservoirs (0,53%) et vecteurs (2,26%). Au sujet du moyen de transmission, une faible proportion des enquêtés (0,08%) avaient cité à la fois la morsure et la griffure. Les connaissances sur le réservoir, le vecteur et le mode de transmission n’étaient pas très variable d’un district à un autre. Le niveau de connaissance de la description de la rage, du réservoir, du vecteur et du mode de transmission selon les districts est consigné dans le Tableau 2.

**Tableau 2:** Connaissance de la définition de la rage, du réservoir, du vecteur et du mode de transmission du virus rabique selon les districts sanitaires, étude réalisée au Tchad, 2015

Définition de la rage	Bénoye (%)	Laoukasssy (%)	Moundou (%)	N’Djaména (%)
Maladie transmise du chien à l'homme	29,16	29,16	48,27	60,46
Maladie liée au cerveau, la tension	47,61	46,29	35,58	31,14
Malnutrition	15,49	15,32	8,57	0,16
Empoisonnement, les esprit	3,79	3,79	1,15	0,00
**Réservoir**				
Chien	100,00	100,00	95,06	94,23
Chat	13,84	5,60	12,85	25,37
Autres animaux domestiques	0,49	0,66	3,95	1,32
Chauves souris	0,16	0,16	0,33	0,66
Ne sait pas	1,81	0,16	0,00	5,27
Renard, hyène, serpent, rat	1,48	0,00	3,95	0,99
**Vecteur**				
Chien	99,84	99,84	99,01	94,23
Chat	19,77	12,36	10,05	28,34
Cutres animaux domestiques	2,47	9,39	0,82	2,31
Chauves souris	0,82	0,99	0,82	1,32
Ne sait pas	1,32	0,16	0,00	0,33
Poulet, rat, pintade	0,00	0,00	0,00	4,94
**Mode transmission**				
Morsure	99,51	100	99,01	98,52
Griffure	4,61	1,15	4,61	8,90
Léchage	0,16	0,00	0,16	0,16
Ne sait pas	0,00	0,00	0,00	0,82

### Connaissances des signes de la rage humaine et animale, du moyen de prévention, du coût de prévention et de traitement

Les caractéristiques prédominantes de la rage humaine connues étaient l’agitation (67%), l’aboiement/comportement semblable à celui du chien (29%) et celles de la rage animale étaient l’agressivité (94%), errance inhabituelle (49,84%), l’agitation (44%). Interrogés sur le principal moyen de prévention de la rage, certains enquêtés (79,78%) avaient mentionné la vaccination des chiens. Pour d’autres, c’était la vaccination du chat (0,49%), l’abattage des chiens errants (5,89%), l’utilisation des pratiques traditionnelles (1,07%). 35,96% des enquêtés avaient défini le coût moyen de prévention de la rage à 7403FCFA (11,23 €) et celui du traitement antirabique était en moyenne 47 667 FCFA (72,66 €) selon l’avis de 51,04% des enquêtés. Ces coûts étaient ignorés respectivement par 58,77% et 47,81%.

### Raison d’arrêt de traitement antirabique, risque de non consultation en cas de morsure et de suspension de traitement antirabique

A la question de savoir quel risque de ne pas consulter une structure de santé en cas de morsure de chiens, 88,4% évoquaient le risque de développer la rage et 5,02% ne connaissaient aucun risque et 0,86% affirmaient qu’il n’y a aucun risque. Le risque d’occasionner le tétanos (3,09%) était aussi évoqué. Le risque de développer la rage suite à l’abandon de la PPE sans avis médical a été évoqué par 70,80% des enquêtés. Sur ce point, 14,62% n’arrivaient pas à donner leur avis.

### Premiers soins à domicile, structures consultées, itinéraire de consultation et délai de consultation en cas de morsure de chiens

Interrogés sur les premiers soins appliqués à domicile en cas de morsure, 47,90% étaient favorables pour les soins traditionnels, 19,69% pour le lavage de la plaie. Il y avait aussi de l’automédication (7,7%), l’utilisation d’alcool (3,46%) mais également aucune action entreprise (20,14%). Le lavage des plaies était la caractéristique des enquêtés de N’Djaména Sud (50,84%) et de Moundou (20,71%) et les soins traditionnels, celles de tous les districts du Logone Occidental. Après les soins à domicile, 72,61% déclaraient se rendre dans une structure de santé. Parmi eux, 27,35% était de N’Djaména Sud, 30,1% de Moundou, 24,65% de Laoukassy et 17,89% étaient de Bénoye. Ceux qui déclaraient continuer avec les soins traditionnels étaient à 44,51% du district de bénoye. Concernant l’itinéraire de consultation en cas de morsure, trois structures étaient citées. Les détails de l’itinéraire de consultation en cas de morsure sont présentés dans le Tableau 3. Quelle que soit la structure, la majorité des enquêtés (93%) exprimaient leur désir de consulter le jour même de l’exposition.

**Tableau 3:** Itinéraire de consultation en cas de morsure selon les enquêtés, résultats de l’étude réalisé dans quatre districts sanitaires au Tchad, 2015

Structures consultées	Première structure consultée	Deuxième structure consultée	Troisième structure consultée
	N= 2428	n= 495	n= 57
Structure de santé humaine (%)	78,50	77,37	50,88
Soins traditionnels (%)	19,02	13,94	17,54
Structure de santé animale (%)	2,48	8,69	31,58

### Cas de morsures des chiens, cas de rage humaine et animale dans les ménages enquêtés et les signes afférents

Certains ménages (4,53%) avaient déclarés 116 cas de morsures au cours des 12 derniers mois parmi lesquels 53,31% étaient les enfants de 0 à 15 ans. 29,31% étaient considérés suspects de rage. Il y avait également 73 cas de rage humaine et 268 cas de rage animale décrits respectivement par 2,28% des et 6,71% des ménages. Les principaux signes évocateurs de la rage humaine étaient l’irritabilité (33,33%), le fourmillement de la plaie (25,93), l’hydrophobie (22,22%), le cri semblable à celui du chien (18,87%) et la disparition de l’animal (16,67%). Ceux de la rage animale étaient le changement de comportement (95,05%), l’agitation (46,48%), la photophobie (18,31%) et la disparition de l’animal (10,56%).

### L’élevage des chiens et la présence des chiens au voisinage des ménages enquêtés

Nos investigations ont montré que 45,55% des ménages élèvent au moins un chien. Certains étaient élevés pour garder la maison (93,22%) ou le troupeau (6,42%). D’autres étaient élevés pour la chasse, pour assistance lors des travaux champêtres, la vente (0,36 %). Les principales raisons pour ceux qui n’en élevaient pas étaient la source de problème que constituerait le chien (40,02%), le manque de moyens financiers (12,25%), la maison non clôturée (9,68%). Pour 60,38% des ménages étaient d’avis pour vendre, manger ou donner les chiens contre les travaux champêtres lorsqu’ils ne peuvent plus assurer leur fonction pour laquelle ils étaient élevés. Interrogés à propos des chiens errants dans le voisinage des ménages, 37% des enquêtés avaient déclaré constater un nombre estimé à 4497 chiens dans leur voisinage.

## Discussion

La taille de notre échantillon est représentative car nous avons tenu compte du taux de non réponse dans le calcul. Face à certains enquêtés, il était nécessaire de traduire le questionnaire en langue locale puis écrire en français les réponses données en ces langues. Dans ces situations, il pourrait avoir des biais d’informations liés à la traduction du questionnaire et à celle des réponses données par les enquêtés. Mais la maîtrise de la langue locale, la parfaite intégration des enquêteurs dans le milieu et l’habitude qu’ils ont dans la collecte des données ont permis de cerner les subtilités de la langue locale et de minimiser ces biais. Dans certains milieux, la considération et le respect d’une décision prise sont liés au sexe et à la position que la personne occupe. Dans le milieu tchadien, Ce sont le sexe masculin et la personne responsable dans le ménage. Notre étude rapporte un nombre d’enquêtés de sexe masculin supérieur à 65,61% et 63,10% des personnes interrogées étaient les chefs des ménages. Nous estimons que les réponses qu’ils ont apportées aux questions posées étaient sensées traduire le comportement des membres du ménage. La connaissance de la morsure de chiens comme moyen de contracter la rage était bonne et celle de la griffure et léchage était médiocre. Cette connaissance n’était pas affectée par le niveau d’éducation et le lieu de résidence des enquêtés. Les soins traditionnels étaient plus pratiqués (47,69) que le lavage immédiat des plaies (19,48%) et l’utilisation de l’alcool (3,46%) en cas de morsures. Et les structures de santé animale étaient moins consultées (2,48%) que les structures de santé humaine et les guérisseurs traditionnels. Il a été constaté que la majorité des personnes exposées au cours des 12 derniers mois étaient des enfants de 0 à 15 ans (53,44%) et que la connaissance des coûts de prévention et de traitement était faible. La proportion 65,61% des enquêtés de sexe masculin ayant participé à notre étude était supérieure celles réalisées à Addis Abeba, au Bangladesh, à Kakamega County ou encore en Tanzanie [9–12] mais en en dessous de celles de Bali et de Dar Town [13, 14]. Comparée à d’autres études, la connaissance de la définition de la rage était inférieure à celle de l’étude de Bali et plus élevée que celle de la Tanzanie [15, 16]. La connaissance du chien comme réservoir, vecteur et de la morsure de chiens comme principal moyen de transmission du virus n’était pas très variable entre les districts sanitaires et était supérieure à 90%. Cette connaissance était similaire aux résultats de l’étude réalisée à Bali et supérieure à celle de l’étude réalisée à Bahir Dar Town [13, 15]. Celle de la morsure comme moyen de transmission était identique aux résultats de Bangladesh, supérieure à ceux de Bahir Dar Town, de Bali et de la Tanzanie et de et inférieurs à ceux d’Addis Abéba qui étaient de 100% [9, 10, 13, 15, 16]. Alors qu’un animal malade de rage peut transmettre le virus aussi bien par morsure que par griffure, la transmission du virus par la griffure était presqu’ignorée méconnue. Globalement la connaissance des moyens de transmission était supérieure aux résultats des études Addis Abbéba en Ethiopie et à Délhi [9, 17]. La forte proportion à consulter le jour de l’exposition était notée aussi à Madagascar, à Abidjan et au Mali [18–21]. Cependant le lavage de la plaie à l’eau et au savon, mesure recommandée pour diminuer le risque de progression du virus, était faiblement observé et inférieur aux résultats de Bahir Dar Town [13]. Les résultats de notre étude montrent que cette pratiques relevant aussi bien de la zone rurale (Laoukassy et Benoye) qu’urbaine (Moundou, N’Djaména Sud) était inférieure à ceux constaté à Gondar, Bahir Dar Town, à Tamil Nadu mais supérieurs à ceux du Kenya [11, 13, 22–24]. La proportion des enfants parmi les personnes était supérieure à l’estimation de l’OMS dans les pays d’Afrique et d’Asie (40%) et aussi aux résultats d’une étude réalisée au Bangladesh [10]. La rage était définie par moins de la moitié des enquêtés comme une maladie transmise par le chien mais les différents noms de la rage en patois montrent une bonne connaissance de la rage et c’est un important atout pour aider davantage à la prise de conscience et à mobiliser les efforts pour la lutte. Il était constaté que les populations ayant un niveau d’éducation élevé décrivaient mieux la rage comme une maladie du chien transmise à l’Homme.

Les points de vue sur la définition de la rage diffèrent d’une zone à une autre. La zone urbaine s’était caractérisée par celle de la maladie du chien transmise à l’homme et la zone rurale était dominée par la maladie liée au cerveau/folie/trouble de comportement/tension, la malnutrition du chien. La faible connaissance de la griffure rejoint la faible attention souvent apporté au cas de morsure superficielle ou unique. La faible connaissance du coût de prévention et traitement n’est pas de nature à permettre aux ménages de déterminer leur choix pour la prévention de la rage qu’elle soit par la vaccination chien, du chat ou la vaccination post-exposition. La plupart des enquêtés était d’avis pour la consultation médicale le premier jour de morsure mais ils doivent entreprendre nécessairement le lavage immédiat des plaies avant de recourir à une structure de santé. Et si ces déclarations sont effectives, elles rassurent sur la prise en charge de la victime sous réserve des pratiques du personnel de santé. La proportion des enquêtés utilisant les soins traditionnels en milieu urbain tchadien était mitigée par rapport aux résultats constatés dans la zone urbaine et rurale de Gondar [23]. Elle était inférieure à celle des études réalisées à Addis Abeba, à Bangladesh, à Bahir Dar Town ou à celle de Gondar [9, 10, 13, 22]. Quelle que soit la zone, les résultats de notre étude étaient supérieurs à ceux du Kwazulu Natal (2%) [25]. L’utilisation excessive de pratiques traditionnelles dans la zone rurale atteste une fois de plus le problème d’accessibilité de l’information sur la rage et du vaccin dans les zones rurales [26]. Il convient aussi de dire qu’il y a un problème d’information et de communication sur les dangers liés à la rage vis-à-vis de ces populations que l’accessibilité au vaccin quand les soins traditionnelles sont également observées en milieu urbain. Ces pratiques vont à l’encontre de la recommandation de l’OMS sur la nécessité d’instituer un traitement médical aux victimes de morsures de chien. Malheureusement elles sont communes aux populations des pays d’Afrique et d’Asie où les cas de morsures sont les plus élevés. En plus de favoriser l’expression clinique de la rage, les pratiques traditionnelles sont un facteur pour la prise en considération du problème de la rage par les décideurs à cause de la sous notification. Le contact avec le vétérinaire après un cas de morsure est indiqué pour le suivi de l’animal et la prise en charge approprié de la victime. Le faible contact avec ces services doit être une préoccupation majeure et l’institution d’un cadre de collaboration des services de santé humaine et animale est exigée pour la gestion des zoonoses et de la rage plus particulièrement. La proportion des enfants exposées est une inquiétude en rapport avec les années de vie perdues dans un contexte où de fortes pratiques traditionnelles, de faible lavage des plaies et de contact avec le vétérinaire pour le suivi de l’animal sans compter la qualité de prise en charge médicale sont constatés. Et nous rappelons que 19% de personnes exposées par une morsure d’un animal malade en mourraient sans PPE [27]. La bonne connaissance de l’agitation comme signe de rage humaine n’est pas à prendre signe critère. L’hydrophobie et l’aboiement comme un chien connus comme signes courants de la maladie sont faiblement rapportés par les enquêtés. Il en est de même pour la rage animale où la photophobie était faiblement rapportée. La rage étant une maladie neurologique, elle pouvait être confondue à d’autres maladies et ne peut être confirmée que par le diagnostic de laboratoire. C’est une raison de plus de demander aux décideurs d’accorder l’importance à la question de la rage à travers les laboratoires de diagnostic aux normes internationales actuelles (l’immunoflurescence). Un besoin de communication et d’appropriation des signes de rage s’impose pour qu’en cas d’exposition et à défaut de prévention primaire qui est la meilleure option (vaccination des chiens), les populations puissent prendre des précautions nécessaires. Pour notre étude, les concessions non clôturées, clôturées sans portail et clôturées ayant un portail mais non fermé sont considérées comme des concessions non clôturées et sont un risque d’exposition à la rage. Ce risque était pour le chien du ménage, ayant une liberté de mouvement qui le mettait en contact à tout moment avec d’autres chiens. Et le risque pour les membres du ménage ainsi que leurs chiens qui pouvaient être surpris à tout moment par un animal malade errant. Cette analyse montre que 74,34% des ménages enquêtés et 76% des ménages qui élevaient au moins un chien étaient concernés. La mobilisation pour la lutte contre la rage est importante et la communication doit être accrue auprès des enfants de façon à les amener à comprendre les différents types d’expositions, les conduites à tenir et de déclarer toutes expositions.

## Conclusion

La communication en matière de la connaissance du chat comme réservoir et vecteur, de la griffure comme moyen de transmission du virus rabique est nécessaire et doit également porter sur le lavage immédiat des plaies et les effets néfastes des pratiques traditionnelles. La connaissance des coûts la prévention et de traitement serait un atout pour un choix optimal à prévenir la rage. Les services vétérinaires sont un maillon non négligeable pour une prise en charge appropriée. Le délai de consultation doit être confirmé par une étude dans nos sites actuels. Les attitudes et pratiques du personnel de santé, le suivi des cas de morsure au sein des populations, le devenir des animaux mordeurs et des personnes exposées au cours d’une surveillance active sur ces sites préciseraient davantage les acquis.

### Etat des connaissances actuelles sur le sujet

- Le chien est connu comme principal réservoir;
- La morsure est le principal mode de transmission;
- Les enfants de 0 à 15 ans sont les plus exposés.

### Contribution de notre étude à la connaissance

- L’itinéraire de consultation montre un problème d’organisation de prise en charge des personnes exposées;
- La faible connaissance de la griffure comme moyen de transmission du virus rabique dans nos sites d’étude;
- Les soins traditionnels sont plus pratiqués que le lavage des plaies comme premiers soins et les guérisseurs traditionnels sont plus consultés que les services vétérinaires en cas de morsure.

## Conflits d’intérêts

Les auteurs ne déclarent aucun conflit d'intérêt.
